# Sleep disturbances in veterans with chronic war-induced PTSD

**DOI:** 10.5249/jivr.v8i2.808

**Published:** 2016-07

**Authors:** Habibolah Khazaie, Mohammad Rasoul Ghadami, Maryam Masoudi

**Affiliations:** ^a^Sleep Disorders Research Center, Kermanshah University of Medical Sciences, Kermanshah, Iran.

## Abstract

Post-traumatic stress disorder is related to a wide range of medical problems, with a majority of neurological, psychological, cardiovascular, respiratory, gastrointestinal disorders, diabetes, as well as sleep disorders. Although the majority of studies reveal the association between PTSD and sleep disturbances, there are few studies on the assessment of sleep disruption among veterans with PTSD. In this review, we attempt to study the sleep disorders including insomnia, nightmare, sleep-related breathing disorders, sleep-related movement disorders and parasomnias among veterans with chronic war-induced PTSD. It is an important area for further research among veterans with PTSD.

## Introduction

Post-traumatic stress disorder (PTSD), classified under Trauma and Stress-Related Disorders in the DSM-IV-TR, is a mental health problem that can occur following experience of a psychologically traumatic event like war, assault, or disaster. The estimated prevalence of PTSD in the general population is 1-9%, among victims with significant trauma the figure is 20–45% and among veterans it is 15–20%.^1^

PTSD symptoms are divided into three clusters: 1) re-experiencing of the traumatic event (e.g., recurrent recollections of the event, including flashbacks and nightmares); 2) avoidance of trauma cues (e.g., avoiding thinking and feeling about the traumatic event); and 3) hyperarousal (e.g., insomnia, hypervigilance).^1,2^

Difficulty in falling and staying asleep and nightmares are symptoms included in the DSM-IV-TR diagnostic criteria for PTSD.^3^ Sleep disturbances in PTSD are core features that are often resistant to first-line treatments. It has been shown that sleep disturbance independently aggravates daytime symptoms and often requires sleep-target intervention. ^4,5^ Sleep disturbances contribute to poor clinical outcomes including poor daytime function, increased suicidality and depression, poorer perceived physical health and increased drug and substance abuse. ^5-7^ Previously, studies showed that patients with chronic psychiatric disorders may be at increased risk of sleep problems and that sleep problems may exacerbate psychiatric disorder symptoms such as anxiety and PTSD.^8,9^

Sleep disturbances are frequent complaints among patients diagnosed with PTSD. Up to 87% of PTSD patients reported subjective sleep disturbances. ^10^ Occurrence of sleep disturbances early after trauma exposure^11^ and when correlated with discriminating sympathetic activity during rapid eye movement (REM) sleep,^12^ have contributed to a greater risk of developing subsequent PTSD up to 1 year later.^11^ Sleep deprivation by acting on the major neuroendocrine stress systems i.e., the autonomic sympatho-adrenal system and the hypothalamic-pituitary-adrenal axis, can sensitize individuals to PTSD.^13^

Studies have demonstrated that although treatment of PTSD may lead to some improvement in sleep disturbances in PTSD patients, this treatment does not produce complete improvement of the sleep disturbances and patients may also stay susceptible to recurrence of symptoms of PTSD. ^14-16^ These facts challenge this concept that sleep disturbances in PTSD are only secondary symptoms that are best treated by treatment of the main disorder. On the other hand, sleep target intervention in PTSD patients results in significant remission in both PTSD symptoms and sleep disturbances. ^17-19^ There are a few studies that have studied sleep disturbances in veterans with PTSD. Previously, Ghadami et al. revealed that veterans with chronic, war-induced PTSD commonly complained of sleep problems, especially insomnia, however, the patients had normal sleep when they were objectively assessed by actigraphy.^9^

Even though many studies have investigated sleep disorders in PTSD, very few have reported on sleep disorders in war-induced PTSD and there has been no review on sleep disorders in chronic, war-induced PTSD. Because of this gap in our knowledge in recognizing specific treatments for sleep disorders, at first, it is necessary to identify sleep disturbance in PTSD patients, especially in veterans with PTSD. So, the aim of this study is review the sleep disturbances in veterans with chronic, war-induced PTSD. We classified these sleep disturbances into several categories: at first we generally characterized sleep difficulties and so forth, then we listed insomnia, nightmares, sleep-related breathing disorders, sleep-related movement disorders and parasomnias.

**1. Characterizing sleep difficulties in veterans with PTSD**

Roszell et al. reported that 91% of 116 Vietnam veterans with a current PTSD diagnosis complained of sleep difficulties.^20^ Also, Neylan et al. when re-analyzing the National Vietnam Veteran Readjustment Survey (NVVRS) indicated that 44% of combat veterans with PTSD reported frequent or very frequent difficulties initiating and maintaining sleep. ^21^ Recently, Plumb et al. revealed that sleep disturbances are common among 375 service members and veterans of operations Enduring Freedom and Iraqi Freedom and reported 89% of the sample as poor sleepers based on the Pittsburgh Sleep Quality Index (PSQI).^22^

The importance of sleep complaints in veterans with PTSD has resulted in several investigations of PTSD-related sleep disturbances using objective measures such as polysomnography and actigraphy. However, investigations have failed to disclose a consistent pattern of sleep architecture abnormalities in PTSD. 

In contrast to the consistent findings of difficulty getting to sleep, difficulty maintaining sleep and nightmares among veterans with PTSD in subjective sleep assessments, the findings based on objective sleep assessments have an apparent discrepancy.

Differences in the findings regarding difficulty getting to sleep and maintaining sleep between the subjective and objective assessments revealed paradoxical insomnia in PTSD patients that is common among these patients. Leskin and colleagues reported that 80% of PTSD patients suffered from paradoxical insomnia.^6^ Also, Ghadami et al., assessed insomnia complaints among 32 veterans with chronic war-induced PTSD, by making a comparison between self-reported PSQI with objective actigraphy recording and found that participants underestimated total sleep time, sleep efficiency as well as number of awakenings and overestimated sleep latency.^9^ These findings revealed that objective sleep parameters do not adversely affect in veterans with chronic PTSD and suggested that in these patients, due to unreliable self-reported sleep disturbance, objective evaluations of sleep disturbance with polysomnography or actigraphy are required.

Results from previous studies of Vietnam veterans with combat-related PTSD have revealed significant differences between veterans with PTSD and controls regarding the sleep measures, as assessed by PSG. In a study by Mellman, et al., they found that recurrent awakenings, threatening dreams, thrashing movements during sleep were the most prevalently reported sleep-related complaints among PTSD patients. Also, in PSG findings, they showed that the PTSD patients, compared to the control group, had reduced sleep efficiency and more awakenings after sleep onset.^23^ In another polysomnographic study by Mellman, et al., in a comparison of individuals with combat-related PTSD, major depression and controls, the authors found decreased sleep efficiency in the PTSD group compared to the other groups.^24^ In sum, Mellman et al. have concluded that sleep maintenance is impaired in veterans with chronic PTSD, and they hypothesized that the sleep disturbances in PTSD patients are the result of more highly aroused behaviors and states, which appear partially conditioned to REM sleep. Consistent with this conclusion, Lavie et al reported that although there were no significant differences between sleep data of patients and controls, veterans with PTSD had significantly higher awaking thresholds that were significantly and positively correlated with depression and anxiety scores.^25^

The study by Woodward et al. on 56 Vietnam combat-related PTSD patients demonstrated that PTSD patients presented a trend toward reduced low-frequency electroencephalogram spectral power during non-rapid-eye-movement (NREM) sleep. These patients presented more beta in REM versus NREM sleep than controls and there was a positive correlation between NREM sleep sigma-band electroencephalogram spectral power and subjective hyperarousal in PTSD patients.^26^ However, other studies have not described differences between veterans with PTSD and controls regarding sleep efficiency and awakenings.^27,28^

Engdahl et al., in a study on 59 males who were exposed to war trauma, showed that those with PTSD had a lower perception of sleep quality than the perceptions of non-PTSD participants. Also, they had a higher percentage of rapid eye movement (REM) sleep and fewer arousals from non-REM sleep than non-PTSD participants. The authors noted that although alterations in REM and arousals characterized PTSD in this sample, with ruling out of comorbid sleep disorders, sleep was clinically similar for PTSD and non-PTSD subjects.^28^ Further, a polysomnographic study on eighteen Vietnam combat veterans with PTSD by Hurwitz et al., revealed no clinically significant sleep disorder or typical pattern of sleep disturbance compared to controls.^27^

In summary, studies on characterizing sleep difficulties in veterans with PTSD have produced inconsistent results, despite the frequent subjective complaints of these patients. Although studies showed that persistent PTSD symptoms following war trauma are associated with sleep disturbances, self-reported poor sleep quality and sleep disturbance do not appear to contribute to PTSD symptoms, beyond the effects of PTSD itself. However, it is important that the complaint of sleep disturbance be considered and objective sleep assessment be done for patients. Increased REM density, reduced REM sleep fragmentation and increased sleep awakening may be sensitive indices of the sleep disturbance in polysomnographic assessments of war-induced PTSD patients.

**2. Insomnia**

Insomnia is a common complaint after traumatic events and causes considerable subjective distress and treatment resistance^29^ and is associated with morbidity among PTSD patients.^30^ Ohayon & Shapiro reported in their community-based study that 41.2% of participants with PTSD had difficulty initiating sleep versus 12.6% of participants without PTSD. In their study, insomnia symptoms were more frequent among PTSD patients than non-PTSD respondents. DSM criteria for insomnia were observed in 39.6% of PTSD respondents, as compared with 6.5% in non-PTSD respondents. Based on this finding, the authors revealed that 60% of PTSD respondents with insomnia complaints had an insomnia disorder, while 20% of non-PTSD insomnia complainers had an insomnia disorder.^31^

The prevalence of difficulty in getting to sleep or sleep-onset insomnia has been reported in Neylan et al.’s study by reanalysis of the NVVRS. They reported that 44% of veterans with PTSD rated themselves as having problems with sleep onset either ‘sometimes’ or ‘very frequently,’ compared to 6% of veterans without PTSD. Further, 91% of veterans with PTSD reported that they had ‘sometimes’ or ‘very frequently’, difficulty maintaining sleep compared to 63% of veterans without PTSD.^21^

Although insomnia is frequent among PTSD patients, several studies have been carried out for a comparison of the subjective and objective sleep assessments in these patients.^27,32-34^ Previously, Ghadami et al. showed that despite the forceful complaints of insomnia in veterans with chronic war-induced PTSD, patients had normal sleep according to actigraphic assessment.^9^ According to the International Classification of Sleep Disorders (ICSD), this controversy between objective and subjective sleep assessments is the hallmark of diagnosis for paradoxical insomnia.^35^

In summary, giving an insight into the severity of the insomnia reported by veterans with PTSD, the findings based on objective measures are mixed with some studies indicating that veterans with PTSD do not have significantly disturbed sleep and low sleep efficiency. Nevertheless, veterans with PTSD frequently complain of insomnia. We suggest that due to unreliable self-reported insomnia in veterans with chronic PTSD, objective sleep evaluations for these patients are necessary. Further study of sleep state perception and objective sleep parameters in veterans with PTSD appears warranted.

**3. Nightmare**

Nightmares of the trauma are one of the defining features of PTSD.^1-4^ In the study of patients who referred to doctors’ practices and hospitals complaining of nightmares, Gupta & Chen, 2006 found that about 32% of patients had PTSD, which was the most frequent diagnosis,^36^ whereas, others reported about 8% nightmare frequency in individuals without PTSD. ^37^ Additionally, in Ohayon and Shapiro’s study, PTSD patients reported more nightmares (19%) compared to the non-PTSD (4%) sample. ^31^

The emotional content of nightmares in veterans with PTSD is varied and contains anger, intense terror, grief, guilt and helplessness.^38^ Dow et al., with the study of Vietnam Veterans with PTSD demonstrated that PTSD patients frequently reported sudden awakenings during sleep that associated with their most striking traumatic experience. ^39^

In reanalyzing the NVVRS, Neylan et al. showed that 52.4% of combat veterans with PTSD complained of nightmares compared with only 4.8% of combat veterans without PTSD. War-zone trauma exposure had shown the strongest correlation with nightmare complaints.^21^

Although high rates of self-reported nightmares in PTSD patients are definite, there are controversies as regards the explanation of the sleep architecture parameters associated with nightmares. Mellman et al., examined sleep events by overnight polysomnography among twenty one combat veterans with PTSD. While 83% of the patient awakenings were related to fear or being startled, only 17% presented trauma-salient dream recall. The authors recommended that because the frequency of threatening dream recall was minimal, these awakenings may be best considered as panic or startle responses.^23^ Ross et al., have suggested that dysregulation of the REM sleep mechanisms may be involved in the PTSD-related disturbances, including insomnia, nightmares and anxiety dreams.^40^

In the study of Vietnam combat veterans with PTSD, Woodward et al., found that only trauma-related nightmares were correlated to increase waking after sleep onset (WASO). They showed that 57% of trauma-related nightmares occurred during REM sleep, 27% occurred during Stage 2 and 10% during Stage 1. However, no associations between nightmare complaints and REM sleep architecture were observed. The authors suggested that increased WASO was specifically related to trauma-related nightmare complaints, and both them are different from normal dreaming, phenomenologically and functionally.^41^

Also, Woodward et al. evaluated PTSD-related hyperarousal during sleep by study of fifty-six unmedicated, nonapneic, Vietnam combat-related inpatients with PTSD and 14 controls who spent three or more nights in the sleep laboratory. In PTSD patients, NREM sleep sigma-band electroencephalogram spectral power revealed a positive correlation with subjective hyperarousal.^42^ According to the findings that nightmares can and do occur throughout the sleep cycle, it was suggested that there may be multiple mechanisms responsible for the elicitation of trauma-related nightmares. 

Numerous studies have investigated the nightmare content experienced by PTSD patients. Mellman et al, demonstrated that although among both PTSD and non-PTSD Vietnam veterans the reporting of disturbing dreams was common, only veterans with PTSD reported dreams of wartime experiences.^23^ Esposito et al., in the study of 18 Vietnam veterans with PTSD showed that half of the dreams included features characteristic of combat, 83% were moderate to highly threatening, fifty-three percent were set in the present, and 79% contained distorted elements. The authors concluded that the target dreams of veterans with PTSD vary regarding to replication of trauma and typically are threatening.^43^

Schreuder et al. assessed 15 veterans traumatized during their military service and 24 civilian war victims. Participants were asked to fill in a questionnaire about their sleep and dreams for four consecutive weeks. The results indicated that posttraumatic nightmares occurred on approximately 20% of total nights and anxiety dreams were reported on 5%. Nightmares and anxiety dreams occurred with equal frequency among veterans and civilian war victims. Nightmares often appeared to be exact replications of the trauma, and replication and repetition were highly correlated. ^44^

Woodward et al., when recording overnight dreams of Vietnam veterans with PTSD in the morning, reported that nightmares about Vietnam were found to be associated with increased awakenings during the night. However, it should be noted that dreams and nightmares are only remembered if they are attended by an awakening.^42^

In a questionnaire-based study which evaluated 35 Vietnam veterans with PTSD and 37 patients with insomnia but not PTSD, Inman et al., showed no differences between the two groups regarding the severity of the insomnia. However, the PTSD group reported more sleep-related anxiety symptoms, such as fear of the dark, fear of going to sleep, having thoughts of the trauma whilst lying in bed and waking up from a frightening dream and then finding it hard to return to sleep.^45^

In summary, it seems that difficulty in initiating and maintaining sleep and frequent nightmares are common complaints among veterans with PTSD. The frequently reported insomnia and nightmares among veterans with PTSD indicated the prognostic importance of sleep disturbances and endorses the presence of them in the criteria for PTSD. Recurrent nightmares have been associated with poor overall sleep quality, depression and heightened risk of suicide. It has been noted that persistent nightmares, those seen in veterans with PTSD, often require targeted treatment interventions.

**4. Sleep-related breathing disorders **

Sleep-related breathing disorders (SRBD) the repetitive episodes during sleep of upper airway closure or obstruction, lead to the stop or decrease of airflow that can force persons to wake from sleep to reestablish their breathing. High frequency (40% – 91%) of SRBD in PTSD versus normal samples (1.2%-3.6%) has been reported in various studies.^10,46,47^ Because of the overall state of sympathetic activation, patients with PTSD referred with SRBD and insomnia.^48^ Krakow et al., reported that crime victims with PTSD reported more nightmares, insomnia, poor sleep quality, leg jerks, and more upper airway resistance syndrome but less snoring.^49^

Despite several reports of more frequent SRBD among patients with PTSD, there is less empirical study to assess sleep respiration among veterans with PTSD. Lavie and Hertz reported faster respiration during sleep among 11 combat neurotics who reported nightmares compared with 9 normal controls.^50^ These findings were confirmed by Woodward et al., in the study of 49 veterans with PTSD and 15 controls. They showed that PTSD patients with trauma-related nightmares showed significantly faster respiration during sleep than PTSD without nightmares. PTSD patients free of nightmares actually breathed more slowly during sleep than controls, and exhibited relative reduction of rate variability during REM sleep, the state from which most nightmares emerge. ^51^

In a case study by Youakim, et al., it was reported that in a 42-year-old Vietnam veteran with PTSD and obstructive sleep apnea, the PTSD symptoms were reduced when the obstructive sleep apnea was treated. ^52^ Consistently, in a study of fifteen PTSD patients, Krakow et al., reported that treatment of SRBD with continuous positive airway pressure (CPAP) was associated with improvements in nightmares and PTSD symptoms. In this study, more patients in the Treatment Group reported improvement in sleep (93% vs. 33%) and in daytime well-being (93% vs. 33%) compared with those in the No-Treatment group. The Treatment Group reported an improvement in nightmares of 85% compared with a 10% worsening in the No-Treatment Group. Nine PTSD patients in the Treatment Group reported a 75% improvement in PTSD symptoms whereas six in the No Treatment Group reported a 43% worsening.^18^ Recently, Yesavage and colleagues, in an observational study of a sample of 105 Vietnam-era veterans with PTSD using overnight polysomnography, reported that 69 % of Veterans with PTSD had an apnea-hypopnea index (AHI) >10, indicative of at least mild OSA.^53^

Williams et al., in a polysomnographic study on one hundred and thirty soldiers diagnosed with PTSD, demonstrated that OSA was diagnosed in 67.3% of subjects. The mean AHI was 24.1±22.8 events/hour among those with OSA. The results also showed that OSA was more frequent among non-injured soldiers than injured soldiers (72.9 vs. 38.0 %, p<0.001). They explained that non-injured soldiers may have suffered from a preexisting undiagnosed sleep breathing problem, which put these individuals at risk for chronic sleep fragmentation, raising the possibility that underlying sleep breathing problem is a risk factor for the PTSD development. Exposure to stressors (even without physical injury) and subsequent sleep fragmentation may have reduced their resiliency and coping capacity, creating a higher potential for PTSD development.^54^

It is difficult to explain the pathophysiological correlation between SRDB and PTSD, in particular because we are unaware whether SRDB is present in trauma survivors before or after they develop PTSD. Regardless of the fact that many studies have shown that there are strong associations between SRDB and insomnia, ^55-58^ it has been shown that PTSD patients with SRDB, report extremely high rates of insomnia complaints.^59^ On the other hand, Series et al. showed that after an experimentally-induced sleep fragmentation, sleep-related breathing abnormalities were frequent among normal sleepers and revealed significant increases in upper airway collapsibility.^60^

Recently, Krakow et al. suggested a bidirectional pathway in which posttraumatic stress-induced sleep fragmentation had a negative effect on the human airway and increased susceptibility to the subsequent development of upper airway collapsibility. This theory indicates a faulty cycle through which PTSD can worsen SDB and through which SDB can worsen anxiety and PTSD symptoms. Based on this theory, they suggested that research will need to focus on the role of sleep in PTSD.^61^

In summary, although studies indicated an association between SRBD and PTSD, the results recommended future investigations of the association between SRBD and PTSD. Nonetheless, the studies had some methodological limitations: random allocation was not assumed to treatment versus no treatment groups; the diagnosis of PTSD was not confirmed by a psychometrically validated interview; and the diagnosis of SRBD was confirmed retrospectively by review of the patient's medical files. The causes and possible mechanisms of the reported association between OSA and PTSD require further investigation, as do the implications for treatment of these two disorders.

**5. Sleep-related movement disorders **

Sleep-related movement disorders such as periodic limb movement disorder (PLMD) are described as repetitive limb movements during sleep and are associated with sleep complaints such as insomnia and excessive daytime sleepiness. Body movements during sleep mainly have a medical origin, not a psychological one, and several diseases e.g. diabetes, renal failure, anemia, are related to PLMD. Limb movements are most frequent in Stages 1 and 2, less frequent in Stages 3 and 4, and mostly absent from REM sleep.^35^

Although, PLMD has more frequent prevalence among PTSD patients than the general population (75% versus 6%),^10^ the relation between PLMD and sleep efficacy among veterans with PTSD has noteworthy findings. Lavie and Hertz revealed a positive correlation between sleep efficiency and movement time among combat neurotics which means that patients who moved less, slept worse. Also, they reported that combat neurotics had higher body movement during Stage 2 sleep compared to controls.^50^ On the other hand, Woodward et al., established reduced sleep movement time (MT) in combat-related PTSD patients compared to controls. There is a strong correlation between reduced MT with more frequent arousals as well as nightmare. The studies’ suggestion that sleep among veterans with PTSD could be related to abridged MT seems to be the opposite of expectations that highly anxious sleep would be characterised by restlessness or agitation. Reduced MT may be due to a freezing state, such as a behavioral response to fear. Woodward et al. recommended that freezing, unlike fight/flight, is an amygdala-triggered, defense response that is not disagreeable with sleep. Sleep with reduced MT and frequent brief awakenings may provide a benefit for survival because raised vigilance is essential for exposure to dangerous environments.^62^

Inman et al., in the study of 35 Vietnam veterans with PTSD and 37 patients with insomnia but not PTSD demonstrated that while there were no differences between the two groups in the severity of the insomnia, the veterans with PTSD were more likely than the non PTSD group to report fragmented waking up, restless legs and excessive body movement during sleep.^45^ Several subsequent studies reported more body movement during sleep for veterans with PTSD compared to control.^23,63,64^

In summary, although several studies illustrated increased limb movement during sleep in veterans with PTSD, it is problematic to identify whether the origins are medical or psychiatric. However, the possibility that PLMD may contribute to insomnia is often endorsed by individuals with PTSD and warrants further investigation. On the other hand, it must be noted that antidepressant medications used in PTSD treatment can increase the incidence of PLMD, and possibly worsen insomnia. The assessment of body movement during sleep across a broader range of veterans with PTSD compared to non-veterans with PTSD and the control of subjects is necessary.

**6. Parasomnia**

Parasomnias are manifestations of CNS activation which may arise due to arousals related to PLMD and SRBD.^30,65,66^ Parasomnias have very complex manifestations: bizarre and vague verbal and/or motor behaviors, such as night terrors, cataplexy, wakeful dreaming, REM sleep behavior disorder (RBD), sleep walking, and sleep paralysis (SP). ^30,65^ Some parasomnias may be sleep-related dissociative disorders where polysomnography shows periods of waking EEG when re-enactment of features of the traumatic experience has occurred. ^59^ There are no large sample size studies on assessment of parasomnias in PTSD or veterans at present, and the main agreement is that major psychological trauma exists in only a minority of cases ^65,66^ of non-REM parasomnias; and that the history of trauma can affect the clinical manifestation of parasomnias. 

RBD and SP are the REM sleep parasomnias that occur in PTSD patients. In RBD there is an absence of REM sleep atonia, which can be related to the acting out of dreams related to traumatic experiences. Husain et al reported a frequency of 56% of PTSD in 27 veterans with RBD.^67^

SP is usually a terrifying symptom that patients are afraid to report for the fear that they will be labeled as crazy. The frequency of SP in the general population is 15% – 40%,^36, 68^ however, recently Gupta et al, reported that 85% of 20 PTSD patients had SP, while only 5% of 20 patients with mood disorders had SP.^69^ Studies illustrate that SP-related hallucinations are bizarre and upsetting and SP-related symptoms in PTSD patients may be described as a sign of schizophrenia. For example, a PTSD patient may wake up with a feeling of having been thrown out of a window.^30^

In summary, given the lack of studies on the assessment of parasomnias among veterans with PTSD, the frequency of parasomnias is unclear. Further study in this field is needed.

## Conclusion

In this review we summarized the literature on the sleep disturbance characteristics of veterans with PTSD. Despite a clear association between PTSD and sleep disturbance, there is a lack of data about veterans with PTSD and existing studies have some methodological limitations including a comparison between the non-veteran PTSD patients with veterans with PTSD. Future research is needed for an evaluation of the baseline mechanism of sleep disturbance among veterans with PTSD; inconsistency between subjective and objective findings in these patients; and especially an assessment of sleep-targeted medical and psychological intervention on the improvement of sleep disturbance as well as PTSD symptoms.
